# Exploring Fecal Microbiota Transplantation for Modulating Inflammation in Parkinson’s Disease: A Review of Inflammatory Markers and Potential Effects

**DOI:** 10.3390/ijms25147741

**Published:** 2024-07-15

**Authors:** Karol Sadowski, Weronika Zając, Łukasz Milanowski, Dariusz Koziorowski, Monika Figura

**Affiliations:** 1 Students Scientific Group NEKON by the Department of Neurology, Faculty of Health Science, Medical University of Warsaw, 03-242 Warsaw, Poland; karol.sadowski@wum.edu.pl (K.S.); weronikazajac182@gmail.com (W.Z.); 2 Department of Neurology, Faculty of Health Science, Medical University of Warsaw, 03-242 Warsaw, Poland; lukasz.milanowski@wum.edu.pl (Ł.M.); dariusz.koziorowski@wum.edu.pl (D.K.)

**Keywords:** fecal microbiota transplantation, Parkinson’s disease, inflammation, microbiota

## Abstract

Parkinson’s disease (PD) is a complex neurodegenerative disorder characterized by numerous motor and non-motor symptoms. Recent data highlight a potential interplay between the gut microbiota and the pathophysiology of PD. The degeneration of dopaminergic neurons in PD leads to motor symptoms (tremor, rigidity, and bradykinesia), with antecedent gastrointestinal manifestations, most notably constipation. Consequently, the gut emerges as a plausible modulator in the neurodegenerative progression of PD. Key molecular changes in PD are discussed in the context of the gut–brain axis. Evidence suggests that the alterations in the gut microbiota composition may contribute to gastroenteric inflammation and influence PD symptoms. Disturbances in the levels of inflammatory markers, including tumor necrosis factor-α (TNF α), interleukin -1β (IL-1β), and interleukin-6 (IL-6), have been observed in PD patients. These implicate the involvement of systemic inflammation in disease pathology. Fecal microbiota transplantation emerges as a potential therapeutic strategy for PD. It may mitigate inflammation by restoring gut homeostasis. Preclinical studies in animal models and initial clinical trials have shown promising results. Overall, understanding the interplay between inflammation, the gut microbiota, and PD pathology provides valuable insights into potential therapeutic interventions. This review presents recent data about the bidirectional communication between the gut microbiome and the brain in PD, specifically focusing on the involvement of inflammatory biomarkers.

## 1. Introduction

Parkinson’s disease (PD) is the second most common neurodegenerative disorder, affecting around 2–3% of the population over 65 years of age. By 2040, the number of confirmed cases is predicted to double, exceeding 10 million worldwide [1]. The clinical manifestation includes motor symptoms caused mainly by the loss of dopaminergic neurons in pars compacta of the substantia nigra (SN) [2]. Three cardinal motor symptoms that the Movement Disorder Society (MDS) considers crucial for diagnosing PD include bradykinesia, tremor at rest, and/or rigidity [3]. Other motor symptoms commonly occurring in PD include hypomimia, postural instability, freezing of gait, akinesia, and micrographia [4]. Numerous non-motor symptoms (NMSs) are also observed in PD, indicating the multi-systemic involvement of the disease. The most common NMSs include olfactory disturbances, constipations, sleep disturbances, depression, and cognitive dysfunction [5]. Loss of cholinergic and glutaminergic neurons and damage within the autonomous nervous system are partially responsible for NMSs [6].

A characteristic feature of PD is the accumulation of α-synuclein, detected in both the central nervous system (CNS) and the peripheral nervous system [2]. α-synuclein is considered to play a vital role in inflammation and immune response. It activates macrophages and specific T cells, releasing proinflammatory cytokines [7]. This has been supported by specific biomarkers of inflammation in the serum, cerebrospinal fluid (CSF), and SN samples obtained from PD patients [8].

PD involves a latent “pre-motor” period of 7–10 years when α-synuclein accumulates in the autonomic nervous system (ANS), with particular involvement of the enteric nervous system (ENS) [9]. The clinical manifestations of these changes are gastrointestinal symptoms, among which constipation is the most prominent. On top of this evidence, Braak et al. proposed a hypothesis of “dual-hit”. It states that the pathology of PD initiates from two locations: the gastrointestinal (GI) tract and the olfactory bulb. In the case of the former, the pathology spreads through the X nerve and propagates to the CNS and nucleus of the X nerve [10,11]. The gut–brain axis, describing the connection of those two systems in the human body, was established. The interplay is not only anatomical, but also via metabolic, humoral, and immune pathways. It has been observed that disturbance in the composition of enteral microbiota can lead to increased inflammation in that area. Thus, fecal microbiota transplantation (FMT) may hypothetically influence some symptoms in patients with PD due to changes in microbiota composition and inflammation [12].

The aim of this work is to review the state-of-the-art data on the role of the microbiota in the inflammatory processes and pathology of PD. It will focus specifically on the effects of enteral microbiota modifications as a potential modulator of PD symptoms.

## 2. Significance of Gut Microbiome Changes in Parkinson’s Disease Patients

The gut–microbiome–brain axis is a connection between the GI tract with its microbiome (bacteria, yeast, and viruses) and the CNS. It is a complex network of communications that impacts the GI tract, movement, and advanced cognitive and emotional functions [12,13]. It achieves this through enteric reflexes, immune activation, entero-endocrine signaling, or changes in intestinal permeability [14].

The intestinal microbiota is composed of microorganisms colonizing the gastrointestinal tract. The large intestine contains the largest amount of microbiota in the body. According to Snder et al., it is approximately 3.8 × 10^13^ number of bacteria and it constitutes 0.2 kg of mass [15,16]. The microbiota takes part in various physiological functions. These include digestion, short-chain fatty acids (SCFAs), and vitamin synthesis, as well as the development and maturation of the immune system [17]. In a state of homeostasis, the dominant species in the gut microbial population belong to the phyla *Firmicutes* (64%) and *Bacteroidetes* (23%) [18]. The changes in microbiota can be affected by the intake of drugs and certain food products, pregnancy, method of child delivery, illnesses and surgeries, among others [19]. If there is a significant change in the microbiota’s constitution or size, a process referred to as dysbiosis occurs [20]. The microbiota starts to differ significantly from an exemplary one, and it can revoke balance, damage the homeostasis, and promote inflammation in the gut. Even though there is no uniform description of dysbiosis that correlates with the development of PD, some unique changes in the microbiota have been observed that promote gut inflammation [13]. For example, a study by Murros et al. indicated that higher levels of *Desulfovibrio* bacteria are detected in patients with PD [21]. The summary of the shifts in the composition of the microbiota of PD patients compared to healthy individuals is presented in Table 1.

Changes in the microbiome directly connected to the inflammation process include imbalance in bacteria belonging to the phylum *Fermicutes.* They manifest as a decrease in bacteria *Roseburia* and *Faecalibacterium* of the *Lachnospiraceae* family that produce SCFA [22]. The SCFA butyrate, propionate, and acetate are metabolites produced by microbes. Their presence in the gut and other organs is influenced by environmental factors such as diet and antibiotic use. SCFAs play a role in regulating the epithelial barrier function and mucosal and systemic immunity through evolutionarily conserved mechanisms involving G protein-coupled receptor signaling or histone deacetylase activity. Notably, butyrate’s anti-inflammatory effects are achieved by directly influencing the differentiation of intestinal epithelial cells, phagocytes, B cells and plasma cells, as well as regulatory and effector T cells [23]. They act as an energy source for intestinal epithelial cells, participate in cholesterol and fatty acid synthesis, and have anti-inflammatory properties [24]. SCFAs also regulate gene expression and therefore affect the intestinal epithelial barrier and function of innate immune cells, such as macrophages, neutrophils, and dendritic cells. Moreover, they cause a reduced synthesis of cytokines and chemokines involved in the recruitment of immune cells to the periphery and in the differentiation of monocytes to macrophages. These include: monocyte chemoattractant protein-1 (MCP-1), chemokine C-C motif ligand (CCL) 3, CCL4, CCL5, chemokine C-X-C ligand (CXCL) 9, CXCL10, and CXCL11 [25]. SCFA production alterations are, therefore, directly related to the development of local inflammation. They may play a role in the pathogenesis of PD and contribute to the manifestation of the gastrointestinal symptoms. Patients with PD showed lower levels of acetic, propionic, and butyric acid in their feces and higher levels of those acids in their plasma. These changes were linked to increased intestinal epithelial barrier permeability, increased immune cell concentration in the periphery, and apoptosis of intestinal epithelial cells [26].

Besides inflammation, the microbiota plays a significant role in drug metabolism, influencing the efficacy of oral drugs. Rekdal et al. reported that *Enterococcus faecalis* and *Eggerthella lenta* produce enzymes that participate in levodopa decarboxylation, decreasing the drugs’ bioavailability. An increase in their levels is also associated with GI tract inflammation [27]. A similar relationship has been observed for *Helicobacter pylori* by Dobbs et al. The authors conducted a retrospective study that included 2105 individuals with H. pylori infection and 9105 unaffected individuals. They concluded that the infected subjects were two to three times more likely to develop PD [28].

Microbiota also has the potential to both trigger and mitigate α-synuclein deposits formation, which is the pathological hallmark of PD. *Helicobacter hepaticus* and curli-producing *Escherichia coli* were identified among the pathogens promoting α-synuclein deposit formation and dopaminergic degeneration in animal models [29,30]. On the other hand, a study by Wang et al. indicated that *Lactobacillus plantarum* DP189 may have an attenuating effect on α-synuclein accumulation in the SN of mice with MPTP-induced parkinsonism [31].These initial results suggest that modifications of gut microbiota may have a disease-modifying effect.

**Table 1:** The summary of the number of family members of the bacteria in Parkinson’s disease (PD) patients compared to healthy controls. ↑ indicates the higher representation of bacteria in PD compared to healthy subjects; ↓ indicates the lower representation of bacteria in PD patients compared to healthy donors. Summarized after Li et al. and Bai et al. [32,33].

Family	Direction of Change
Bifidiobacteriaceae	↑
Rikenellaceae	↑
Ruminococcaceae	↑
Lactobacillaceae	↑
Verrucomicrobiaceae	↑
Christensenellaceae	↑
Erysipelotrichaceae	↓
Faecalibacterium	↓
Lachnospiraceae	↓
Prevotellaceae	↓

## 3. Fecal Microbiota Transplantation

**Figure 1:**
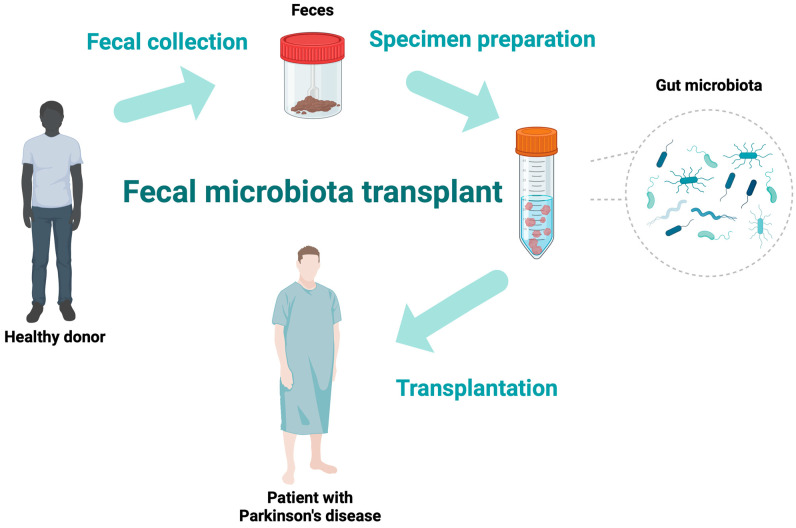
The schematic diagram of the fecal microbiota transplantation process.

FMT is a method of transferring fecal contents (the whole gut microbiota) from a healthy individual into the gastrointestinal tract of a patient to achieve a therapeutic advantage [34,35]. This can be obtained with the delivery of microbiota during colonoscopy or gastroscopy, by a nasojejunal tube, or the oral administration of capsules containing microbiota. Figure 1 illustrates the exemplary process of FMT. FMT has become increasingly well-known in recent years due to its effectiveness in treating many gastrointestinal disorders. A milestone in FMT research occurred in 2013 when the Food and Drug Administration (FDA) accepted FMT as a routine treatment for recurrent *Clostridium difficile* infections (RCDIs) [34]. Due to its efficacy in the treatment of RCDIs, studies have been performed to implement this treatment in other diseases involving gut dysbiosis. Due to the presence of gastrointestinal symptoms in the progress of various neurological disorders, such as multiple sclerosis, epilepsy, autism, and PD, FMT has been evaluated as a method to ease the symptoms of these disorders, with promising initial results [36,37].

## 4. Inflammatory Process Alterations in PD

Inflammatory processes in PD can be detected in the SN, but they can also occur in the GI tract. According to an analysis of colonic biopsies of 19 PD patients conducted by Davos et al., a significant alteration in the mRNA expression levels of proinflammatory cytokines assessed by a real-time PCR study showed considerable variability in the expression of proinflammatory cytokines among PD patients [38]. The pathogenesis of inflammation in the intestinal wall could be linked to altered microbiota. It influences permeability of the intestinal wall, which allows the spread of pathogens and toxins from the intestine to CNS [39,40,41,42]. This may also trigger the accumulation of α-synuclein in the ENS. Louveau et al. suggested that the dural sinuses may be a potential route for transporting immune cells, connecting the gastrointestinal tract to the CNS [43]. It is difficult to determine whether increased gut–blood barrier permeability is a cause or effect of PD. Studies have shown that patients with inflammatory bowel disease (known to increase gut permeability) have a higher risk of developing PD [12,44]. Microbial cell structures and pattern-inflammatory recognition receptor signaling pathways activate inflammatory processes. The higher representation of *Proteobacteria* of the genus *Ralstonia* signify a greater exposure to endotoxins, such as bacterial lipopolysaccharide (LPS)-binding protein. It also leads to amplified levels of proinflammatory cytokines, e.g., interferon-γ (IFN-γ), interleukin (IL) 1β, IL-6, or tumor necrosis factor α (TNF-α) [12,24,45]. Additionally, IL-1β and C-reactive protein (CRP) are increased in the stools of PD patients [41]. An alteration of numerous inflammation-related markers has been observed in PD. Increased levels of IL-1β, IL-6, and TNF-alpha in the serum are induced by LPS, which may originate from an altered gut microbiota [46]. The greater exposure to endotoxins and proinflammatory cytokines may promote α-synuclein transcription, increasing protein synthesis [47]. Miranda-Morales et al. additionally reported that the methylation of the α-synuclein promoter region affects α-synuclein expression. Therefore, the enteral microbiome is an epigenetic factor due to its role in DNA methylation [48].

To date, there are no clear correlations between the concentrations of inflammatory markers in plasma, CSF, and PD. Kharpenko et al. reported elevated serum IL-1β and IL-6 and reduced IL-1RA levels in the PD group. In both CSF and the serum, inflammatory markers exhibited distinct patterns. Elevated TNF-α in CSF was linked to a rapid progression of PD, while increased IL-1β was observed in the serum. A lower IL-6 level was linked to a prolonged PD duration. High serum IL-10 levels were associated with anxiety, depression, lack of tremor, and late-onset PD. PD patients with mild cognitive impairment had lower serum TNF-α levels than the controls. Additionally, serum IL-1β, IL-6, and IL-10 levels were positively correlated with CSF biomarkers [49]. Wijeyekoon et al. also reported no correlation between the concentrations of cytokine in plasma and CSF [50].

### 4.1. TNF-α

TNF-α is a major regulator of inflammatory responses and is known to be involved in the pathogenesis of inflammatory and autoimmune diseases [51]. High levels of TNF-α have been detected in CSF and post-mortem brains of PD patients [52,53]. This may suggest that TNF-α mediates neuronal damage and, thus, is a potential target for PD treatment [54,55,56]. Iwaoka et al. showed increased levels of TNF-α and IL-1β in PD patients’ CSF than in the controls [57]. King et al. and Koziorowski et al. reported similar findings: they showed increased TNF-α levels in blood samples [58,59]. The altered microbiota also induces higher concentrations of TNF-α and IFN-γ in PD plasma patients. Lin et al. reported a correlation between *Bacteroides* and the plasma level of TNF-α and a correlation between *Verrucomicrobia* abundance and plasma concentrations of IFN-γ [60]. A 2016 meta-analysis of nine studies involving 809 patients and meta-analysis including 2654 patients performed by Qin et al. in 2022 revealed elevated peripheral TNF-α concentrations in patients with PD [61,62]. Similarly, Schorder et al. showed increased concentrations of TNF-α in patients with PD [63]. In contrast, Kim et al. and Alrafiah et al. among others indicated no difference between healthy populations and PD patients, or even lower levels in PD patients’ serum [49,64,65,66,67,68]. In a paper by Carvey et al., no neurodegeneration was observed after administering a single 20 ng dose of TNF-α in the SN in a rat model [69]. Similarly, delivering 500 pg of TNF-α did not cause the degeneration of dopaminergic neurons within 7 days. However, a dose of TNF-α 100-400 times higher resulted in dopaminergic cell loss in the SN [69,70]. Overall, acute TNF-α expression at typical levels appears to be non-toxic for the SN dopaminergic neurons. The difference between chronic and acute exposure may be of significance. De Lella Ezcurra et al. investigated the impact of chronically upregulated low levels of TNF-α. [70]. They reported a progressive neurodegenerative effect with the clinical manifestation of forelimb akinesia and a distinct inflammatory response in the rat brain after TNF-α injection [70]. These results indicate that the long-term expression of proinflammatory levels of TNF-α cause toxic effects in the SN [69].

**Figure 2:**
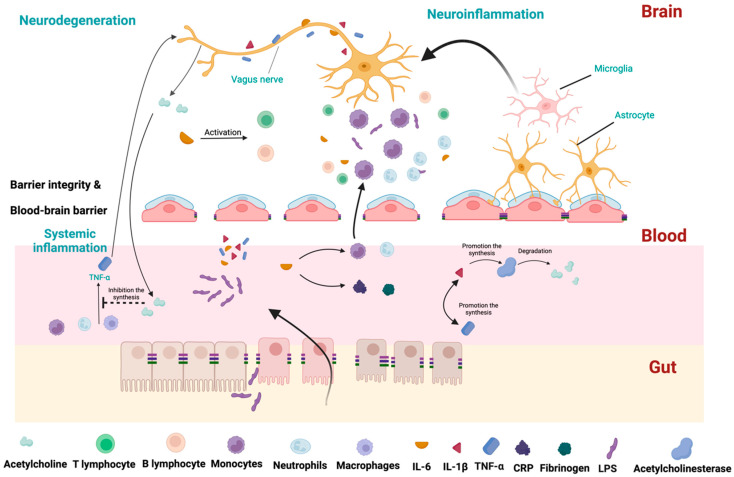
The crucial pathways representing the interplay between gut microbiota and inflammation. Due to increased intestinal permeability, LPS can translocate to blood circulation. LPS increases levels of IL-1β, IL-6, and TNF-α. TNF-α activates cells to release IL-1β. To control this response, TNF-α stimulates the X nerve’s afferent branch, which conveys this signal to the CNS. Interleukin-6 (IL-6) stimulates the production of acute-phase proteins, such as CRP and fibrinogen. IL-6 promotes the migration of neutrophils and monocytes to the inflamed tissue. IL-6 also regulates activity of T and B lymphocytes and promotes the development of regulatory T cells, which help to dampen the immune response and prevent excessive inflammation. IL-1β promotes the synthesis of acetylcholinesterase, which decreases acetylcholine and promotes inflammation. In the periphery, IL-1β production leads to TNF-α production (and vice versa). In the brain, IL-6 stimulates T and B lymphocytes, leading to the activation of microglia and astrocytes. Monocytes can cross the blood–brain barrier, which also promotes inflammation. The motor branch of the X nerve releases acetylcholine in the periphery, which interacts with macrophages and other cytokine-releasing cells. It inhibits the release of proinflammatory cytokines, particularly TNF-α.

The cholinergic anti-inflammatory pathway should also be considered to understand the contribution of TNF-α in PD pathogenesis. This pathway regulates the innate immune response after infections. During the response, TNF-α is produced mainly by macrophages, but also by B lymphocytes and NK cells. It prolongs the inflammatory response by activating other cells to release IL-1β. However, this initial beneficial response can be detrimental if it lasts longer or is exacerbated. To control this response, TNF-α stimulates the X nerve’s afferent branch, which conveys this signal to the CNS, as presented in Figure 2. In turn, the efferent branches of the X nerve release acetylcholine. The neurotransmitter interacts with nicotinic acetylcholine receptors expressed on macrophages and other cytokine-releasing cells. This activates intracellular signal transduction, which inhibits the release of proinflammatory cytokines, particularly TNF-α. This negative feedback circuit regulates the inflammatory response, maintaining homeostasis [71]. Initial studies showed that FMT could effectively decrease the concentration of TNF-α [72,73,74].

### 4.2. IL-1β

**Figure 3:**
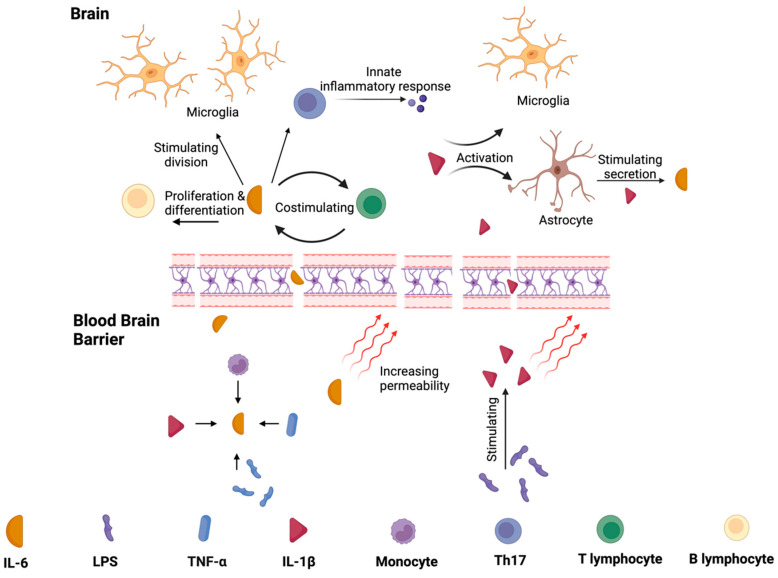
The functions of IL-1β and IL-6 in peripheral tissues and central nervous system. Monocytes, IL-1β, LPS, and TNF-α promote the synthesis of IL–6, which increase the blood–brain barrier’s permeability. It stimulates the division of microglia and the differentiation of B lymphocytes. IL-6 production also leads to T lymphocytes production (and vice-versa). These pathways promote inflammation. LPS increase the level of IL-1β, and thus, IL-1β activates microglia and increases the level of IL-6 via stimulating astrocytes to release this cytokine.

IL-1β is a major cytokine activating proinflammatory signaling pathways in peripheral tissues and the brain [75]. Increased levels were detected in CSF and the post-mortem brains of individuals with PD [52,76]. IL-1β promotes acetylcholinesterase synthesis, decreasing acetylcholine availability and promoting inflammation [77]. Additionally, IL-1β increases TNF-α production [77,78,79]. These data are summarized in Figure 3. Depending on the study and the material assessed, the amount of IL-1 β was mainly described as higher in PD [57,62,65,80,81]. On the other hand, some researchers did not report differences in IL-1β expression in PD compared to the control group [59,68]. Bacterial LPS stimulates its production; so, one might hypothesize that altering the microbiota would modulate its levels. Studies confirmed that FMT could effectively decrease the concentration of IL-1β [82,83]. These are also described in more detail in the chapter “The Efficacy of FMT” [74].

### 4.3. IL-6

IL-6 is a signaling molecule that plays a crucial role in inflammation by stimulating the production of acute-phase proteins, such as CRP and fibrinogen. IL-6 also plays a role in recruiting immune cells to the site of infection or tissue damage by promoting the migration of neutrophils and monocytes to the inflamed tissue. Additionally, IL-6 has anti-inflammatory properties, mainly regulating the activity of T and B cells. It can also promote the development of regulatory T cells, which limit the immune response and prevent excessive inflammation [84]. Most studies focusing on the role and presence of IL-6 in serum have demonstrated altered levels of IL-6 in PD patients compared to the control group, indicating its involvement in inflammatory processes in PD [49,58,62,66,81]. However, other authors suggest a lack of correlations or even lower concentrations of IL-6 in PD [59,65,67]. The effects of IL-1β and IL-6 are summarized in Figure 3.

A paper by Chen et al. indicated that high concentrations of IL-6 in plasma were linked to an augmented risk of developing PD [85]. Bessler et al. have demonstrated that in vitro L-dopa treatment of peripheral mononuclear cells derived from PD patients (with augmented baseline production of IL-6) further increased the production of this cytokine [86]. Numerous studies have researched the association between IL-6 serum concentration and the clinical status of the disease [87,88,89]. Hasegawa et al. observed a negative correlation between the production of IL-6 in LPS-stimulated peripheral blood mononuclear cells of PD patients [90]. This hypothesis also complies with the study by Selikhova et al., where higher serum levels of IL-6 in patients with rapid PD progression compared to patients with slower progression were described [91]. To date, studies have shown that FMT could effectively decrease the concentration of IL-6 [92]. Bottigliengo et al. concluded that higher concentrations of IL-6 were linked with the earlier onset of the disease [93]. In addition, a study by Zhao et al. in mice models of parkinsonism also indicated the effectiveness of FMT in lowering concentrations of IL-6 [74]. The study included the induction of gut microbiota dysbiosis in mice by rotenone administration. Later, the animals were divided into two groups, FMT and placebo without further intervention. It was shown that FMT effectively restored the gut microbiome. It led to improvement in both gastrointestinal issues and motor impairment. It also reduced IL-1β, IL-6, and TNF-α serum levels [74].

### 4.4. IL-10

IL-10 is an anti-inflammatory cytokine that prevents the degeneration of dopaminergic neurons in SN [94]. A meta-analysis by Qin et al. included 2654 patients with PD who presented an increased concentration of IL-10 [62]. Similarly, a study by Rentzos et al. involving 60 patients with PD revealed increased the levels of IL-10 in the PD group versus the healthy control group [95]. Similar results were reported in studies by Rathnayake et al., Brockmann et al., and Qin et al. [62,96,97]. In contrast, some other studies suggest a lack of correlations or only weak tendencies for correlation between PD and elevated IL-10 concentration or even lower concentration [59,63,68]. Koziorowski et al. reported no difference in the concentration of IL-10 in the serum between the PD group and the control group [59]. Similar results in CSF were obtained by Schroder et al. [63]. Rocha et al. presented contrary results, mainly that the plasma concentration of IL-10 was lower in the PD patients’ group [68]. A meta-analysis of studies on inflammatory markers in PD indicates that patients with PD have an increased level of IL-10 [62]. FMT modulates different immune pathways, leading to an anti-inflammatory effect. This process involves activating different types of immune cells, including CD4+ T cells and antigen-presenting cells (APCs), producing IL-10. Several studies on mice models have demonstrated decreased GI inflammation after FMT, primarily attributed to an increased IL-10 production [98,99]. Korolkova et al. showed similar findings in human subjects [100]. However, the specific impact of FMT on IL-10 production in individuals with PD has yet to be investigated.

### 4.5. TGF-β

TGF-β is a cytokine essential in the modulation of inflammatory processes. TGF-β regulates the differentiation and function of B and T lymphocytes, controls the magnitude and type of immune responses against microbes, and helps to maintain tolerance against antigens [101]. A meta-analysis performed by Chen et al. demonstrated that PD patients presented increased levels of TGF-β in the brain and CSF [102,103]. In contrast, a paper by Santaella et al. reported a similar level of TGF-β in CSF in PD patients compared to a healthy population [104]. It was demonstrated that FMT inhibits the TGF-β signaling pathway and attenuates inflammation in rat models of PD [105]. A study by Li et al. implied that FMT might influence this pathway by the modulation of intestinal microbiota, leading to a reduction in inflammatory markers induced by LPS [106]. The abovementioned results from animal models may suggest that a similar modulation may be achieved in PD patients.

### 4.6. NF-κB

NF-κB is the transcription factor involved in pro-inflammatory processes by modulating the survival, activation, and differentiation of innate immune system cells and T cells [107,108]. Studies suggested that the crosstalk between the gut microbiome and the NF-κB signaling pathway substantially contributes to the aging process and associated pathologies [109]. Consequently, targeting the NF-κB pathway by manipulating the gut microbiome may be a promising intervention for age-related disorders [109]. Li et al.’s paper showed a significant reduction in NF-κβ signaling pathway after FMT in mice models [110]. This was supported by a study by Jing et al., demonstrating that FMT downregulated IL-1β/NF-κB signaling in the spinal cord and NF-κB signaling in mice gut [111]. The importance of this pathway in PD pathogenesis may be highlighted by the fact it is activated following intestinal bacterial infection. Host cells initiate the intracellular NF-κB signaling pathway to prompt a response of antibacterial immunity and maintain the integrity of the intestinal barrier [112].

## 5. The Effectiveness of FMT in PD

GI microbiota disruption is increasingly recognized as a key factor for PD pathogenesis. However, most studies that suggest reduced inflammation after FMT have come from animal models. Zhao et al. confirmed decreased levels of inflammatory markers TNF-α, IL-1β, IL-6, nitric oxide synthase inhibitor (iNOS), (cycloosygenase) COX-2, and β-actin occurring after FMT in mice, leading to reduced dopaminergic neuron damage [74]. Furthermore, according to the authors, FMT reduces the amount of LPS in the gut, serum, and SN, inhibiting specific inflammatory pathways [74]. Similarly, Zhang et al. assessed the amount of mRNA of inflammatory markers and showed a reduced amount of IL-1β in a mouse model after FMT [113]. In addition, Sun et al. demonstrated that FMT can effectively minimize neuroinflammation and control components of the TLR4/TNF-α signaling pathway in the brain and intestine of PD mice [114].

Individual studies have supported the efficacy of FMT in patients with PD. These are primarily based on the reduction in the scores in clinical scales measuring NMS, neurological examination, microbiota composition changes, and inflammatory markers present in the serum and gut [115,116,117,118,119]. The GUT–PARFECT trial has yielded promising results. It is a single-center randomized, double-blind, placebo-controlled trial including 46 patients with PD (24 patients receiving own microbiota–placebo and 22 patients receiving microbiota from healthy donors). There was an improvement in the healthy donor-FMT group with a decrease of 5.8 points in MDS-Unified Parkinson’s Disease Rating Scale (MDS-UPDRS) score compared to a 2.7 points reduction in the placebo group (*p* = 0.0235). The greatest difference was present in the 6 to 12 months interval. The findings from the radiopaque pellets tests show that the group receiving treatment experienced a decelerated advancement of constipation compared to the group administered a placebo [115]. The notable contrast in MDS-UPDRS motor scores between the treatment groups emerging between the 6- and 12-month mark suggests a potential disease-modifying effect rather than solely symptomatic improvement [115].

Segal et al. conducted a study involving six PD patients, and they reported an improvement in motor symptoms, NMS, and constipation [118]. Similarly, Xue et al. observed improved scores on the Pittsburgh Sleep Quality Index, Hamilton Depression Rating Scale, Hamilton Anxiety Rating Scale, Parkinson Disease Questionnaire, Non–motor symptoms questionnaire, and UPDRS part III in 15 patients after FMT [120]. Huang et al. analyzed the occurrence of motor symptoms following FMT, indicating that limb tremor declined after the procedure. They also showed that not only the frequency, but also the intensity of constipations decreased [119]. The results of the available studies are summarized in Table 2. Despite the encouraging findings of FMT, there are still obstacles to overcome: creating consistent methods for stool preparation, determining the most effective delivery methods, building an extensive registry of donors and recipients, and determining the most efficient method of administration (capsules versus enteral administration with tube or endoscope) [121].

### FMT Efficacy and “Body First versus Brain First” Hypothesis

The effectiveness of FMT among PD patients may vary. An important factor that should be included is the “brain first vs. body first” theory introduced by Borghammer et al. [122]. It makes a distinction between patients with early involvement of peripheral nervous system (“body first” PD), with early pronounced dysautonomia and REM behavior disorder (RBD), and “brain first” PD, where the CNS is involved early in the course of the disease. The finding was supported by extensive imaging studies, including not only brain functional and morphological assessment, but also GI tract evaluation with ^11C^-donepezil PET, computer tomography-based measurement of colonic volume, and colonic transit [123]. These domains differ significantly between “brain first” and “body first” patients, confirming a more pronounced peripheral involvement in PD patients with RBD. While data on microbiota composition in those subtypes are lacking, one can hypothesize that differences should be present. Cirstea et al. reported that compositional and metabolic alterations of gut microbiota in PD are highly associated with its dysfunction, which is more pronounced in the “gut first” subtype [124]. Patients with “gut first” PD could therefore benefit more from FMT in terms of reduction in constipations, improvement in colonic transfer, or improvement in levodopa absorption. The reduction in the local inflammation of the GI tract could also decrease the propagation of the inflammatory processes to CNS. This group may therefore benefit more in terms of the neuroprotective effect of FMT. In the “brain first” PD subtype, early FMT could, on the other hand, lessen the level of involvement of the autonomic nervous system and decrease the peripheral spreading of the disease. Further research is needed to confirm this hypothesis. The subtype of PD should, however, be included in studies assessing efficacy of FMT on PD patients, as the effect may vary between subtypes and the results may be inconclusive without proper distinctions.

## 6. Conclusions

Despite the vast investigation of the PD pathophysiology, its origin etiology remains elusive. However, recent findings underscore a correlation between heightened inflammation and PD pathogenesis, with the gut microbiota possibly playing a pivotal role. Studies have indicated that the modulation of the gut microbiota of PD patients may a have potential impact on inflammatory pathways. FMT emerges as a potential therapeutic approach to reinstating gut homeostasis, thereby mitigating inflammation and decelerating disease progression.

Ongoing clinical trials investigating the interplay between PD and inflammation hold promise for advancing our comprehension of this intricate process, unveiling potential directions for therapeutic interventions. The evaluation of FMT remains promising, with substantial potential in regulating the inflammatory effect of the microbiota and providing optimism for clinical outcomes in PD.

## Figures and Tables

**Table 2:** Summary of most important studies on FMT in the therapy of Parkinson’s disease. FMT, Fecal microbiota transplantation; PD, Parkinson’s Disease; UPDRS, Unified Parkinson’s Disease Rating Scale; PSQI, Pittsburgh Sleep Quality Index; HAMD, Hamilton Depression Rating Scale; HAMA, Hamilton Anxiety Rating Scale; PDQ-39, Parkinson’s Disease Questionnaire; PD NMS, Parkinson’s Disease Non-motor Symptoms Scale; NMSQ, Non-motor Symptoms Questionnaire.

Study	Number of Participants	Main Outcomes	Results	Main Conclusions
2024, Bruggeman et al. [115]	46 patients with PD randomly assigned to FMT: 22 from healthy donors; 24 auto-stool transplantation.	Decrease in MDS-UPDRS III scores	Mild but long-lasting improvement in motor symptoms	FMT has potential to modulate the gut microbiome and serve as a therapeutic approach for PD
2023, DuPont et al. [116]	12 patients with constipation and mild to moderate PD. Patients randomly assigned to placebo and FMT groups. Patients received orally lyophilized FMT product in capsules.	Increase in microbiota diversity indices. Change in NMS and PDQ-39 scales	Only temporary improvement for motor symptoms. Significant improvement in gastrointestinal symptoms. Increase in the diversity of gut microbiota.	Changes in microbiota diversity contributed to decrease in gastrointestinal symptoms and improved subjective motor and non-motor symptoms
2021, Kuai et al. [117]	11 patients with PD and constipation. FMT during colonoscopy.	Hoehn–Yahr stage, UPDRS, NMSQ, and constipation scales decreased; lactulose H2 breath tests	Increase in microbiota abundance and diversity. Decrease in constipation frequency and severity.	FMT may prove effective in decrease in the severity of gastrointestinal symptoms due to improvement in the quality and quantity of intestinal microbiota
2021, Segal et al. [118]	6 patients with median age of PD onset 52 years and PD median duration 5 years. FMT during colonoscopy.	Decrease in PSQI, HAMA, HAMD, PD NMS, PDQ-39	Improvement in both motor and non-motor scores, including gastrointestinal symptoms. Only one patient experienced adverse effects from the procedure, which were not severe and did not require further treatment.	FMT administered via colonoscopy is a safe procedure and leads to an improvement in the symptomology of PD
2019, Huang et al. [119]	71-year-old male patient with the onset of PD 7 years before with severe constipation	Reduction in constipation and tremor in lower extremities	Decrease in the severity of constipation. Temporary improvement in motor symptoms for at least 2 months; the severity of motor symptoms decreased.	FMT may have a positive effect on the clinical manifestations of PD

## Data Availability

No new data were created or analyzed in this study.
